# Monitored Anesthesia Care by Sedation-Trained Providers in Acute Stroke Thrombectomy

**DOI:** 10.3389/fneur.2019.00296

**Published:** 2019-03-28

**Authors:** Diana E. Slawski, Hisham Salahuddin, Linda Saju, Julie Shawver, Andrea Korsnack, Gretchen Tietjen, Thomas J. Papadimos, Alicia C. Castonguay, Vieh Kung, Richard Burgess, Syed F. Zaidi, Mouhammad A. Jumaa

**Affiliations:** ^1^Department of Neurology, University of Toledo Medical Center, Toledo, OH, United States; ^2^Department of Neurology, ProMedica Toledo Hospital, Toledo, OH, United States; ^3^Department of Anesthesiology, University of Toledo Medical Center, Toledo, OH, United States

**Keywords:** stroke, mechanical thrombectomy, anesthesia, sedation, provider, thrombectomy

## Abstract

**Background:** Mechanical thrombectomy (MT) for ischemic stroke can be performed under local anesthesia (LA), conscious sedation (CS), or general anesthesia (GA). The need for monitoring by anesthesia providers may be resource intensive. We sought to determine differences in outcomes of MT when sedation is performed by an anesthesia team compared to sedation-trained providers.

**Methods:** We performed a retrospective analysis on patients who were screened by a pre-hospital stroke severity screening tool and underwent MT at two stroke centers. Baseline characteristics, time metrics, sedatives, peri-procedural intubation, complications, and outcomes were recorded. Good outcome was defined as modified Rankin score of ≤2.

**Results:** We analyzed 104 patients (sedation-trained provider = 63, anesthesia team = 41) between July 2015 and December 2017. In the sedation-trained provider group, four patients required intervention by an anesthesia team. There were no differences in patients receiving LA (sedation-trained provider 24% vs. anesthesia team 27% *p* = 0.82), CS (70 vs. 63%, *p* = 0.53), or GA (6 vs. 10%, *p* = 0.71) between groups. Sedation-trained providers were more likely to use only one drug during the procedure (62 vs. 34%, *p* = 0.009). The rate of procedural complications (9.5 vs. 4.5%, *p* = 0.48), good outcome (56 vs. 39%, *p* = 0.11), and mortality (22 vs. 24%, *p* = 0.82) was similar between groups. Sedation by provider type did not predict functional outcome or mortality at 3 months.

**Conclusions:** Sedation-trained providers are capable of delivering appropriate sedation without compromising patient safety. The use of “as needed” anesthesia teams for MT may have considerable effect on resource allocation and cost.

## Introduction

Modalities of anesthesia for acute stroke thrombectomy have significantly evolved over the last several years. Traditionally, standard practice involved performing mechanical thrombectomy (MT) under general anesthesia (GA) to reduce patient movement and consequently lower the risk of complications. With advances in clot retrieval technology, the need for GA was reevaluated and conscious sedation (CS) emerged as a safe, evidence-based alternative (1–5).

The reduced complexity of anesthesia care in MT presents an opportunity for cost control, and may allow smaller stroke centers to provide advanced care as the demand for MT increases. A recent survey in Nordic countries reported that 37% of anesthesiology departments who were equipped to handle MT cases were unable to provide rapid response teams for MT due to limited personnel availability, and 74% of respondents indicated that MT cases are “occasionally” managed by non-anesthesia personnel (6). In this same study, 16% of anesthesia departments provided only GA services for MT, and did not staff cases under CS (6). Similarly, a study of practices in tertiary stroke centers in Spain revealed that anesthesia for MT was managed by non-anesthesia providers in 21% of the hospitals surveyed (7).

The evolution of sedative medications and clot retrieval devices has improved the safety and efficacy of MT, such that the interventionist may readily direct both sedation and revascularization without detriment to the patient. This model of interventionist-directed sedation is already used in elective as well as emergent procedures across subspecialties. In this study, we evaluate outcomes in acute stroke patients who underwent MT with a sedation-trained team vs. a dedicated anesthesia team.

## Methods

### Patients and Protocols

This retrospective review of our prospective Rapid Arterial oCclusion Evaluation (RACE) MT registry was conducted with approval from the institutional review board, and a waiver of informed consent was granted. Additional data from this study is available by request from the corresponding author. Of the 307 patients who underwent MT at our two centers between July 2015 and December 2017, 104 patients were triaged as RACE alert patients and included in this analysis. The method of pre-hospital assessment and triage at our institutions has been published previously (8), but in brief, consists of a grading scale used by emergency medical services to identify patients with potential large vessel occlusions and transport them directly to a thrombectomy-equipped stroke center if the RACE score is >5. Patients were treated at a comprehensive stroke center (CSC) or a primary stroke center (PSC) with thrombectomy capability. Both are level one trauma centers with anesthesia teams in-house. Eligibility for MT was determined by an Alberta Stroke Program Early CT Score (ASPECTS) >6 on non-contrast head CT and evidence of large vessel occlusion on CT angiogram. For patients presenting beyond 8 h of symptom onset, CT perfusion imaging was required to demonstrate small core infarct and salvageable penumbra. Triage and selection protocols were identical at both institutions.

MT was performed using stent retrievers (Trevo, Solitaire) and/or aspiration. All procedures were staffed by two experienced neuro-interventionalists (MJ and SZ) with certification in neuro-critical care, sedation, and advanced cardiac life support. At our institutions, neuro-interventionalists are certified in critical care and have moderate sedation privileges. For nursing staff, hospital policy mandates successful completion of an online training course in sedation and a passing score on competency evaluation. Sedation-trained nurses are supervised by physicians in the neuro-interventional suite.

MT was performed under LA or CS, unless there were strong indications for GA, such as airway compromise or severe patient agitation. Protocols for the presence of anesthesia teams varied per institution. At the PSC, dedicated anesthesia providers [anesthesiologist or certified registered nurse anesthetist (CRNA)] do not staff MT procedures unless called for by the neuro-interventionalist in the event of patient decompensation. Sedation plans are determined by the interventionist. Sedation-trained nurses perform interval airway and neurologic assessments, monitor vitals, and administer all medications. Fentanyl and midazolam are typically used to achieve CS, wherein the patient is still able to respond to verbal commands and maintain an open airway with minimal discomfort from the procedure. At the CSC, the protocol for anesthesia involvement generally requires the presence of a CRNA and supervising anesthesiologist, whose role is to formulate the sedation plan in conjunction with the interventionist, administer all sedatives, and monitor patient stability. However, in cases where anesthesia personnel are unavailable, the neuro-interventionalist assumes responsibility for sedation planning and medications are given by sedation-trained nurses with the goal of achieving moderate sedation. At both centers, any patients who are initially given moderate sedation but subsequently develop airway compromise or combativeness are intubated and placed under GA.

Upon completion of MT, all patients were taken directly to the neuro-intensive care unit and underwent CT imaging at 24 h to evaluate for intracerebral hemorrhage. Intra- procedural and post-procedural protocols are identical at both centers.

### Data Collection and Variables

Data included baseline demographics, treatment variables, complications, and outcomes. We used procedure logs and anesthesia notes to collect additional information regarding presence or absence of anesthesia personnel, need for intubation, sedatives used, and intra-procedural complications during MT. In addition, we recorded initial blood pressure on arrival to the intervention suite, three reads prior to reperfusion, three reads after reperfusion, and ranges for blood pressure while in the neuro-interventional suite. For patients who achieved reperfusion (TICI 2b and TICI 3), the time to reach target systolic blood pressure per institutional protocol of <140 mm Hg was recorded. Aspiration pneumonia was recorded if symptoms developed within 72 h of MT. Incidence of post-procedural intubation was recorded if it occurred within 24 h of MT. Successful revascularization was defined as Thrombolysis in Cerebral Infarction (TICI) score of ≥2b. Symptomatic intracranial hemorrhage (sICH) was determined by post-procedural evidence of hemorrhagic transformation on imaging with concurrent increase in National Institute of Health Stroke Scale (NIHSS) score by 4 or more points. All NIHSS and modified Rankin scale (mRS) assessments were performed by certified nurse practitioners or stroke neurologists during the admission and at 90-day follow-up.

### Statistical Analysis

All statistical analysis was performed using software R: A language and environment for statistical computing; EZR version 1.32. Continuous variables were compared using the Student *t*-test or the Mann-Whitney test, and categorical data with the Fisher exact test where appropriate. A univariate and multivariable logistic analyses were performed to determine predictors of a good clinical outcome at 3 months and mortality. A sensitivity analysis removing posterior circulation occlusions was also performed.

A repeated measure ANOVA analysis was used to compare blood pressures in time and standard deviation, coefficient of variation, and successive variation in each group was performed to reflect blood pressure variability between the two groups.

## Results

We analyzed 104 patients between July 2015 and December 2017 at one PSC (47 patients) and one CSC (57 patients). Sixty-three patients underwent MT with sedation guided by the neuro-interventionalist, and 41 patients had a dedicated anesthesia team present at the beginning of the procedure. In the sedation-trained provider group, four patients required conversion to general anesthesia by an anesthesia team. Two of these patients were moving and agitated despite sedation, one patient developed airway compromise, and one patient required intubation to facilitate direct carotid access. Baseline characteristics were comparable between the two groups (Table 1).

**Table 1:** Baseline demographics, stroke characteristics, and procedural time metrics.

	**Total (*n* = 104)**	**Sedation trained providers (*n* = 63)^**∧**^**	**Anesthesia team (*n* = 41)**	***P*-value**
**BASELINE CHARACTERISTICS**				
Age				
Mean ± SD	72.6 ± 13.9	72.6 ± 13.1	72.6 ± 15.1	0.805
Range	30–97	40–96	30–97	
Female (%)	59 (56.7)	33 (52.4)	26 (63.4)	0.314
Atrial fibrillation	48 (46.2)	26 (41.3)	22 (53.7)	0.234
Hypertension	84 (80.8)	48 (76.2)	36 (87.8)	0.203
Hyperlipidemia	60 (57.7)	35 (55.6)	25 (61)	0.686
LDL (mean ± SD)	81.3 ± 36.1	83.5 ± 39.4	78.0 ± 30.7	0.757
Diabetes Mellitus	28 (26.9)	17 (27)	11 (26.8)	1.0
HbA1c (mean ± SD)	6.1 ± 1.1	6.0 ± 1.1	6.2 ± 1.2	0.374
Smokers	25 (24.0)	16 (25.4)	9 (22)	0.816
CAD	30 (28.9)	16 (25.4)	14 (34.1)	0.380
**SELECTION SCAN**				
CT	29 (27.9)	14 (22.2)	15 (36.6)	0.123
CTA	59 (56.7)	40 (63.5)	19 (46.3)	0.106
CTP	16 (15.4)	9 (14.3)	7 (17.1)	0.784
**STROKE CHARACTERISTICS**				
Admission NIHSS (median, IQR)	16 (12.8–21)	16 (12.5–21)	17 (13–20)	0.864
ASPECTS (median, IQR)	9 (8–9)	9 (8–9)	9 (7.8–9)	0.986
Left sided	55 (52.9)	36 (58.1; *n* = 60)	19 (46.3)	0.314
IV-tPA	57 (54.8)	37 (58.7)	20 (48.8)	0.420
Proximal ICA only	2 (1.9)	1 (1.6)	1 (2.4)	1.0
ICA terminus	26 (25.0)	18 (28.6)	8 (19.5)	0.358
M1	45 (43.3)	25 (39.7)	20 (48.8)	0.421
M2	29 (27.9)	18 (28.6)	11 (26.8)	1.0
Basilar/PCA only	2 (1.9)	1 (1.6)	1 (2.4)	1.0
Concomitant Proximal ICA	13 (12.5)	8 (12.7)	5 (12.2)	1.0
**TREATMENT METRICS (median min; IQR)**				
LKN to arrival time	74 (45–252)	73 (45–194.8)	74 (53–369)	0.519
Door to groin	64.5 (48–77.5)	67 (52–77.5)	60.5 (45.8–75.8)	0.443
Door to recanalization	91 (79.8–114)	91 (80.8–114.5)	91.5 (71.3–103.5)	0.333
LKN to groin puncture	144 (108–314)	147 (109–244)	135 (102.8–443.3)	0.84
Procedure time	24 (17.8–36.5)	25 (16.8–48.5)	21 (18–33)	0.315
LKN to recanalization (TICI 2b or 3)	194 (133.5–317.8)	196 (135–287.3)	173 (129.5–521.8)	0.957
Only Aspiration	33 (31.7)	20 (31.7)	13 (31.7)	1.0
Stent retriever	63 (60.6)	39 (61.9)	24 (58.5)	0.838
Successful Revascularization	86 (82.7)	51 (81)	35 (85.4)	0.608

∧ *4 patients in the sedation trained provider group converted to general anesthesia*.

Patients in the sedation-trained provider group were equally likely to be intubated at the end of the procedure compared to when an anesthesia team was available (6.3 vs. 9.8%; *p* = 0.709). A similar proportion of patients received LA only in both groups (23.8% in sedation-trained provider group, 26.8% in anesthesia team group; *p* = 0.8). Patients in the anesthesia provider group were more likely to receive multiple drugs to achieve sedation compared to patients in the sedation-trained provider group (39.0 vs. 14.3%; *p* = 0.005). Fentanyl was the preferred first-line agent in most cases in both groups.

**Figure 1:**
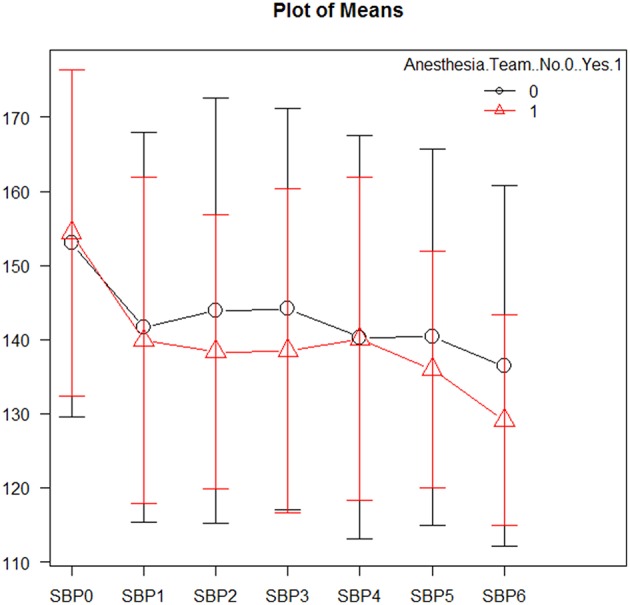
Mean systolic blood pressures during mechanical thrombectomy. Patients in the sedation-trained provider group are represented in black, and patients in the anesthesia team group are represented in red. SBP0 represents systolic blood pressure on arrival to the interventional lab. SBP1-3 are measurements taken prior to revascularization, while SBP4-6 are measurements taken after revascularization.

Mean systolic and diastolic blood pressures were comparable between the two groups (see Figure 1 and Supplementary Material). Vasopressive medications were used equally in both groups (4.8 vs. 4.9%; *p* = 1.0). Successive variation was greater post-intervention in the sedation-trained group for both systolic (15.3 vs. 7.0; *p* = 0.001) and diastolic blood pressure (13.3 vs. 5.2; *p* = 0.003), however coefficients of variation and time to target systolic BP (<140 mmHg) post-intervention were similar (6.8 vs. 4.0; *p* = 0.556). Analysis of blood pressures between groups is displayed in Supplementary Table 3.

More cases of SAH were present in the sedation-trained provider group, although the difference was not statistically significant (5 vs. 1; *p* = 0.399). There was one case of sICH in both groups. Post-procedure intubation, aspiration pneumonia, arrhythmia, number of days on a ventilator, and use of vasopressors were similar between both groups (Table 2). The overall procedural complication rate was numerically higher in the sedation-trained group (9.5 vs. 4.9%, *p* = 0.48). Rates of good clinical outcome (mRS ≤2) (55.6 vs. 39%; *p* = 0.112) and mortality at 3 months (22.2 and 24.4%; *p* = 0.816) were similar in the sedation-trained provider and anesthesia team groups, respectively (Table 2). No difference was observed between the analyses when posterior circulation occlusions were excluded from the cohort.

**Table 2:** Patient outcomes, details pertaining to anesthesia, and procedural complications.

	**Total (*n* = 104)**	**Sedation trained providers (*n* = 63)^**∧**^**	**Anesthesia team (*n* = 41)**	***P*-value**
**CLINICAL OUTCOMES**				
90 day mRS ≤2 (*n* = 104)	51 (49.0)	35 (55.6)	16 (39.0)	0.112
90 day mRS ≤1 (*n* = 104)	36 (34.6)	25 (39.7)	11 (26.8)	0.210
90 day mortality	24 (23.1)	14 (22.2)	10 (24.4)	0.816
Median discharge NIHSS; IQR	4 (1.8–8)	4 (1–8)	4.5 (2–9.5)	0.738
Median improvement NIHSS; IQR	9 (5–13.3)	9 (5–15)	8.5 (5.3–12.8)	0.856
**FINAL ANESTHESIA TYPE**				
Local anesthesia	26 (25.0)	15 (23.8)	11 (26.8)	0.818
Conscious sedation	70 (67.3)	44 (69.8)	26 (63.4)	0.527
General anesthesia	8 (7.7)	4 (6.3)	4 (9.8)	0.709
**NUMBER OF DRUGS USED**				
0	26 (25.0)	15 (23.8)	11 (26.8)	0.818
1	53 (51.0)	39 (61.9)	14 (34.1)	0.009
Multiple	25 (24.0)	9 (14.3)	16 (39)	0.005
2	17 (16.4)	5 (7.9)	12 (29.3)	0.006
3	4 (3.9)	1 (1.6)	3 (7.3)	0.32
4	4 (3.9)	3 (4.8)	1 (2.4)	1.0
**ANESTHESIA-RELATED FACTORS**				
Aspiration pneumonia	11 (10.6)	7 (11.1)	4 (9.8)	1.0
Any procedural intubation	8 (7.7)	4 (6.3)	4 (9.8)	0.709
Pre-procedural intubation	3 (2.9)	0 (0)	3 (7.3)	0.059
Intra-procedural intubation	5 (4.8)	4 (6.3)	1 (2.4)	0.646
Post-procedure intubation	6 (5.8)	4 (6.3)	2 (4.9)	1.0
Vasopressors^*^	5 (4.8)	3 (4.8)	2 (4.9)	1.0
Any arrhythmia^*^	28 (27.0)	15 (23.8)	13 (31.7)	0.498
Atrial fibrillation^*^	23 (22.1)	12 (19)	11 (26.8)	0.469
Sinus bradycardia^*^	4 (3.9)	2 (3.2)	2 (4.9)	0.646
Paced^*^	1 (1.0)	1 (1.6)	0 (0)	1.0
No. of days on ventilator	0.88 ± 2.6	0.81 ± 2.5	1.0 ± 2.9	1.0
**PROCEDURAL COMPLICATIONS**				
Wire perforation	1 (1)	1 (1.6)	0 (0)	1.0
sICH	2 (1.9)	1 (1.6)	1 (2.4)	1.0
Subarachnoid hemorrhage	6 (5.8)	5 (7.9)	1 (2.4)	0.399
All complications	8 (7.7)	6 (9.5)	2 (4.9)	0.475

∧ *4 patients in the sedation trained provider group converted to general anesthesia*.

* *Denotes trait was recorded during mechanical thrombectomy*.

We performed a univariate logistical analysis and determined clinical predictors of a good clinical outcome. When adjusted for confounders, only NIHSS (OR 0.869 CI 0.795–0.948, *p* = 0.001) was a significant predictor of a good clinical outcome. Likewise, adjustment for confounders revealed that NIHSS (OR 1.17 CI 1.06–1.29, *p* = 0.002) and M1 middle cerebral artery location of occlusion (OR 0.271, CI 0.09–0.778, *p* = 0.015) were significant predictors of mortality at 3 months. Sedation type used and sedation by provider type were not significant predictors of a good clinical outcome or mortality at 3 months.

## Discussion

### Summary of Major Findings and Trial Comparisons

In this study, we evaluated outcomes in patients who underwent MT with an anesthesia provider or sedation-trained neuro-interventionalist and nursing staff. Stroke severity and baseline comorbidities were well-balanced in the patient groups. Our data illustrate that selected patients treated by sedation-trained staff have comparable rates of good functional outcome and mortality at 90 days as those patients treated by anesthesia personnel. Similarly, revascularization rates were similar between the two groups, suggesting that technical success was not compromised when the interventionalists assumed the role of sedation provider. Although not directly comparable, the outcomes of our study groups are in line with the CS arms of landmark MT trials outlined in Table 3 (1, 2, 4, 9). Data from our present study suggests that sedation-trained staffs are capable of safely managing MT in cases where GA is not required.

**Table 3:** Conscious sedation arms of recent MT clinical trials.

	**Rate of revascularization**	**sICH**	**Vessel perforation**	**Aspiration pneumonia**	**mRS 0–2 at 90 days**	**Mortality at 90 days**
Current study, STP arm^*^, *N* = 63	81%	1.6%	1.6%	11.1%	55.6%	22.2%
GOLIATH CS^**^ arm *N* = 63	60.3%	1.6%	0%	ND	52%	12.7%
SIESTA CS arm *N* = 77	80.5%	ND	2.6%	3.9%	18.2%	24.7%
AnStroke CS arm *N* = 45	88.9%	6.7%	2.2%	15.6%	40%	24.4%
HERMES CS arm *N* = 561	76%	4%	2%	8%	50%	13%

* *STP, sedation-trained provider; group includes 4 patients who required conversion to general anesthesia*.

** *CS, Conscious sedation*.

The patients included in this study were homogenously triaged by EMS for presenting symptoms suggestive of a large vessel occlusion that would be amenable to urgent thrombectomy. Activation of the “RACE” alert system for these patients ensures the quickest possible door-to-recanalization times (91 min in this cohort). Unlike inter-hospital transfers, this model is not always conducive to drawing off-site personnel such as home-call anesthesia teams or anesthesia staff engaged in ongoing procedures at the time of need. Additionally, unlike inter-hospital transfers, triage of this group of patients is uniformly dependent on distance to the receiving hospital and not on other patient comorbidities.

### Hemodynamic Management

Management of blood pressure in acute ischemic stroke has been shown to have significant effects on long-term functional outcomes. Blood pressure management during endovascular therapy was similar between sedation-trained providers and anesthesiologists in the current study. An intra-procedural fall in mean arterial pressure (MAP) of 10–40%, low pre-revascularization MAP, and high blood pressure variability post-procedure are reported risk factors for poor neurologic outcome (10–12). Various studies have reported a risk of general anesthesia-associated hypotension during neurointervention (10, 13, 14); however the results of a *post-hoc* analysis of the SIESTA trial challenged this finding (15). Rates of any procedural intubation were similar between the two groups, indicating that blood pressure differences in our study were unlikely to be related to intubation.

Compared to a study by Whalin et al. our cohort had much lower rates of vasopressor use (4.8 vs. 52%), although this is likely due to differences in institutional protocols regarding intra-procedural blood pressure management (14). Reduction in MAP of >40% was seen in 11.3% of patients in the sedation-trained group and 7.3% in the anesthesia group (*p* = 0.736), indicating that this likely did not have a significant effect on clinical outcomes between the two groups.

Although post-procedure successive variation for blood pressure was significantly different between the two groups, other measures of blood pressure variability post-procedure (standard deviation, coefficient of variation, time to target BP post-revascularization) were similar.

### Complications and Safety

Clinical trials have shown that procedural complications are infrequent in patients who receive CS for MT (1, 2). In our study, we report no significant differences between patients in the sedation-trained provider group and anesthesia provider group for rates of aspiration pneumonia, sICH, and SAH. There was a trend toward more frequent SAH in the sedation provider group, though only one of these patients had experienced wire perforation of a vessel. The complication rates in our sedation-trained provider group were similar to those reported in the CS arms of recent clinical trials (Table 3).The sedation-trained provider model may draw criticism for the potential to delay treatment in cases where an anesthesia team must be called when a difficulty is encountered. Though the rate of conversion from CS to GA is relatively infrequent (3), there may be an advantage to having anesthesia personnel in the neuro-interventional suite for a more rapid conversion. In our study, four patients in the sedation provider group required conversion and intubation by anesthesia team members, with a median time from page to intubation of 10 min. In the anesthesia provider group, one patient required conversion to GA with a procedural delay of 5 min. It is unclear whether this potential delay would significantly affect outcomes.

### Selection Bias

Patients with complex comorbidities and greater stroke severity pose a challenge in anesthetic management, particularly if the interventionist is directing sedation and performing thrombectomy simultaneously. It is possible that in our cohort there was a selection bias favoring sicker patients to receive sedation from anesthesia providers. We sought to identify characteristics that would present more challenging features for anesthesia care, such as baseline stroke severity, need for intra-procedural vasopressors, and intra-procedural arrhythmia. By these measures, our results show that the distribution of patients with more advanced needs was equal between provider groups. This suggests that patients with complex needs were equally likely to be treated by sedation-trained providers as anesthesia providers. Furthermore, our similar rates of good outcome between groups implies that experienced interventionists are equipped to manage patients of varying levels of complexity without compromising outcomes. A controlled study with randomized distribution of CS candidates of varying complexity to either provider type would reduce the impact of selection bias.

### Cost Reduction and Shifting Models of Care

The indications for MT are expanding and allow for more aggressive pursuit of revascularization. The publication of DAWN and DEFUSE 3 (11, 16) have mobilized a shift toward later reperfusion with an extended time window, and this will undoubtedly increase the need for MT. As more patients become eligible for this treatment, cost and efficiency must be prioritized. The sedation-trained provider model can deliver on reducing costs by allowing patients to be safely sedated and monitored by capable staff, with an overall simplified care structure and lower resource utilization. The growth of MT may also lead to increased reliance on PSCs for endovascular care, and in this case the sedation-trained provider model may prove to be more efficient for smaller stroke centers with limited anesthesia personnel.

While patients with severe agitation, Glasgow coma scale <8, difficult airways, compromised posterior circulation, or an inability to protect their airway may benefit from GA (17), our study demonstrates that the majority of cases may be effectively managed by non-anesthesia providers administering CS. The comfort level of some nurses has been reported as variable in providing care to acute ischemic thrombectomy patients; nonetheless, this can be resolved with appropriate training and support (18).

Anesthesia by sedation-trained providers may be a new feature in stroke care; however, there is extensive literature on current practices utilizing sedation-trained providers in uncomplicated, minimally invasive cardiac procedures. A survey of cardiac EP physicians found that while 71% of providers prefer a dual model of anesthesia professionals and nurses, the majority of non-general anesthesia procedures such as ablations were staffed by sedation nurses only (19). Under acute conditions, such as myocardial infarction requiring PCI, the cardiologist frequently directs patient sedation unless the patient begins to decompensate, at which point the anesthesia team is called (20, 21). The role of sedation-trained providers in emergent, non-invasive cardiac procedures may serve as a model for stroke thrombectomy care.

### Limitations

This study is limited by its retrospective design. There is potential for bias in the distribution of patients between groups, as elaborated in the discussion. The protocols for MT were different at the PSC and CSC; however, the groups were similarly triaged by EMS and were of comparable acuity level. Finally, the sample size for this study is relatively small and limits the ability to detect differences between groups. As the number of posterior circulation cases in our cohort was limited (*n* = 2), future studies are needed to investigate the applicability of a sedation-trained provider model to this subpopulation of patients. Despite these limitations, we believe this study provides encouraging preliminary data for the safety of anesthesia by sedation-trained providers in MT. By drawing attention to this clinical question, we hope to promote discussion among providers to determine whether this practice can be safely integrated in routine MT procedures.

## Conclusion

As MT has made great strides in safety and technical efficiency, attention is now shifting toward streamlining treatment models. Conscious sedation for MT has been shown to be a safe alternative to general anesthesia, and our study suggests that there is no difference in outcome when sedation is administered by a trained provider vs. anesthesia provider. Our study, while retrospective, illustrates that this model may be safe and feasible with appropriate sedation training of both interventionist and nursing staff, and may be capable of delivering maximum speed and efficiency without compromising patient safety. Multi-institutional randomized controlled studies will provide greater evidence and will be a vital step toward the widespread implementation of the most appropriate model for anesthesia care.

## Data Availability

The datasets generated for this study are available on request to the corresponding author.

## Ethics Statement

This study was approved by the University of Toledo IRB. A waiver of consent was used.

## Author Contributions

DS participated in study design, data collection, writing manuscript. HS participated in data collection, data analysis and writing manuscript. LS participated in data collection, data analysis and critical manuscript revisions. JS participated in data collection and critical manuscript revisions. AK participated in data collection, analysis and critical manuscript revisions. GT, TP, AC, VK, and RB participated in data interpretation and critical manuscript revisions. SZ and MJ participated in study design, data analysis and interpretation, and critical manuscript revisions.

### Conflict of Interest Statement

The authors declare that the research was conducted in the absence of any commercial or financial relationships that could be construed as a potential conflict of interest. The handling editor declared a past co-authorship with one of the authors AC.
