# Supplementary material for: Monitored Anesthesia Care by Sedation-Trained Providers in Acute Stroke Thrombectomy
Source: Front Neurol. 2019 Mar 28;10:296. doi: 10.3389/fneur.2019.00296 (PMC6447680; doi:10.3389/fneur.2019.00296)
Supplement: Supplementary file 1 [file Data_Sheet_1.docx]

**ONLINE SUPPLEMENT**

Additional analyses for the following manuscript:

Monitored anesthesia care by sedation-trained providers in acute stroke thrombectomy.

**Supplementary Table I.** Medications administered during mechanical thrombectomy with sedation-trained providers or anesthesia providers.

**Supplementary Table II.** Medications administered to patients who underwent mechanical thrombectomy by conscious sedation or general anesthesia, regardless of provider type.

**Supplementary Table III.** Blood pressure data for patients undergoing mechanical thrombectomy with sedation-trained providers or anesthesia teams.

**Supplementary Figure I.** Mean diastolic blood pressures during mechanical thrombectomy.

|  | **Sedation-trained provider, no conversions**  **N=59** | **Anesthesia team, including conversions**  **N=45** | **p-value** |
| --- | --- | --- | --- |
| **No sedation** | 15 (25.4%) | 12 (26.7%) | 1.0 |
| **Fentanyl** | 44 (74.6%) | 30 (66.7%) | 0.392 |
| **Midazolam** | 5 (8.5%) | 11 (24.4%) | 0.0308 |
| **Dexmedetomidine** | 0 (0%) | 2 (4.4%) | 0.185 |
| **Propofol** | 0 (0%) | 14 (31.1%) | < 0.0001 |
| **Multiple drugs** | 5 (8.5%) | 18 (40%) | 0.0002 |

**Supplementary Table I.** Medications administered during mechanical thrombectomy with sedation-trained providers or anesthesia providers. Patients who began the procedure under interventionist sedation but required conversion to general anesthesia are included in the anesthesia team group in this table. Overall p-value < 0.0001.

|  | **Total** | **Conscious (or no) sedation**  **N=96** | **General anesthesia**  **N=8** | **p-value** |
| --- | --- | --- | --- | --- |
| **No sedation** | 27 | 27 (28.1%) | 0 (0%) | 0.108 |
| **Fentanyl** | 76 | 69 (71.9%) | 7 (87.5%) | 0.679 |
| **Midazolam** | 17 | 11 (11.5%) | 6 (75%) | 0.0002 |
| **Dexmedetomidine** | 2 | 1 (1.0%) | 1 (12.5%) | 0.149 |
| **Etomidate** | 1 | 0 (0%) | 1 (12.5%) | 0.077 |
| **Propofol** | 15 | 9 (9.4%) | 6 (75%) | < 0.0001 |
| **Multiple drugs** | 24 | 16 (16.7%) | 8 (100%) | < 0.0001 |

**Supplementary Table II.** Medications administered to patients who underwent mechanical thrombectomy by conscious sedation or general anesthesia, regardless of provider type. Dexmedetomidine and propofol for conscious sedation were always administered by an anesthesia professional. Overall p-value (excluding multiple drugs) is < 0.0001.

|  | **Sedation provider** | **Anesthesia provider** | **p-value** |
| --- | --- | --- | --- |
| BP ranges |  |  |  |
| Highest SBP | 167.9 ± 23.0 | 161.9 ± 23.6 | 0.247 |
|  | 165 (154 – 180) | 160 (148.5 – 175.5) |  |
| Lowest SBP | 117.5 ± 21.1 | 121.6 ± 18.6 | 0.328 |
|  | 117.5 (101.25 – 130.5) | 120 (105 – 130) |  |
| Highest DBP | 89.4 ± 18.1 | 88.1 ± 22.7 | 0.719 |
|  | 86 (78 – 100) | 82 (78.75 – 100) |  |
| Lowest DBP | 68.8 ± 18.5 | 71.2 ± 16.1 | 0.414 |
|  | 66 (60-78) | 70 (59.5 – 80.75) |  |
| Repeated Measures ANOVA  (Graph of means) |  |  |  |
| SBP |  |  | 0.618 |
| DBP |  |  | 0.427 |
| BP metrics |  |  |  |
| MAP Fall more than 40% | 7/63 (11.1) | 3/38 (7.3) | 0.736 |
| Time to SBP < 140  (minutes) | 6.8 (0-14.3) | 4 (0-24) | 0.556 |
| Target BP within 10 minutes | 36 / 63 (57.1) | 25 / 41 (60.9) | 1.0 |
| Any SBP reading < 140 mm Hg prior to recanalization | 27 / 63 (42.9) | 16 / 41 (39.0) | 0.839 |
| Systolic BP |  |  |  |
| Standard Deviation |  |  |  |
| Pre Recanalization | 14.1 ± 9.5 | 11.4 ± 8.1 | 0.203 |
| Post Recanalization | 9.9 ± 7.0 | 7.7 ± 7.7 | 0.087 |
| Coefficient of Variation |  |  |  |
| Pre Recanalization | 10.0 ± 7.0 | 7.8 ± 5.6 | 0.202 |
| Post Recanalization | 7.4 ± 5.8 | 5.6 ± 5.1 | 0.142 |
| Successive Variation |  |  |  |
| Pre Recanalization | 15.1 ± 17.5 | 13.2 ± 19.4 | 0.133 |
| Post Recanalization | 15.3 ± 22.7 | 7.0 ± 8.1 | 0.001 |
| Diastolic BP |  |  |  |
| Standard Deviation |  |  |  |
| Pre Recanalization | 8.8 ± 6.6 | 11.9 ± 9.8 | 0.137 |
| Post Recanalization | 9.0 ± 10.3 | 6.0 ± 6.3 | 0.136 |
| Coefficient of Variation |  |  |  |
| Pre Recanalization | 11.0 ± 8.5 | 14.4 ± 10.2 | 0.092 |
| Post Recanalization | 11.4 ± 12.0 | 8.1 ± 7.0 | 0.207 |
| Successive Variation |  |  |  |
| Pre Recanalization | 11.9 ± 11.0 | 12.4 ± 13.6 | 0.813 |
| Post Recanalization | 13.3 ± 20.4 | 5.2 ± 6.3 | 0.003 |

**Supplementary Table III.** Blood pressure data for patients undergoing mechanical thrombectomy with sedation-trained providers or anesthesia teams. SBP= systolic blood pressure; DBP = diastolic blood pressure. Successive variation of blood pressure measurements post-recanalization was significantly greater in the sedation provider group. Otherwise, there were no significant differences between groups.


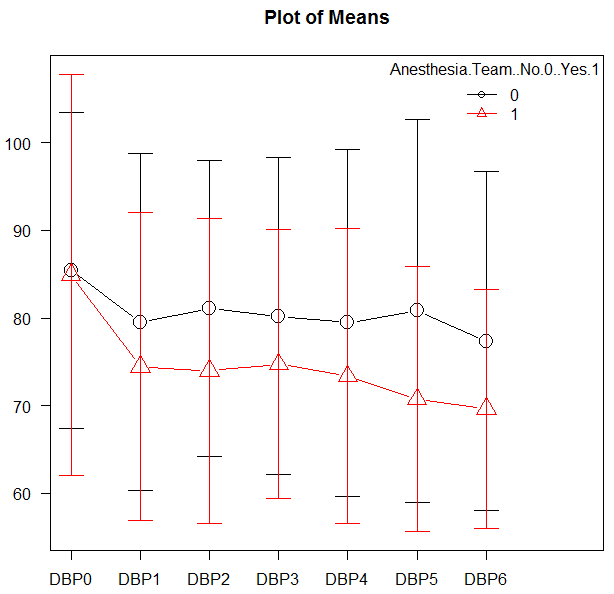


**Supplementary Figure I.** Mean diastolic blood pressures during mechanical thrombectomy.

Patients in the sedation-trained provider group are represented in black, and patients in the anesthesia team group are represented in red. DBP0 represents diastolic blood pressure on arrival to the interventional lab. DBP1-3 are measurements taken prior to recanalization, while DBP4-6 are measurements taken after recanalization.
